# Levels of Emotional Awareness: Theory and Measurement of a Socio-Emotional Skill

**DOI:** 10.3390/jintelligence9030042

**Published:** 2021-08-19

**Authors:** Richard D. Lane, Ryan Smith

**Affiliations:** 1 Department of Psychiatry, University of Arizona, 1501 N. Campbell Ave., Tucson, AZ 85724, USA; 2 Laureate Institute for Brain Research, 6655 South Yale Ave., Tulsa, OK 74136, USA; rsmith@laureateinstitute.org

**Keywords:** emotion, levels of emotional awareness, cognitive development, socio-emotional skills

## Abstract

Emotional awareness is the ability to conceptualize and describe one’s own emotions and those of others. Over thirty years ago, a cognitive-developmental theory of emotional awareness patterned after Piaget’s theory of cognitive development was created as well as a performance measure of this ability called the Levels of Emotional Awareness Scale (LEAS). Since then, a large number of studies have been completed in healthy volunteers and clinical populations including those with mental health or systemic medical disorders. Along the way, there have also been further refinements and adaptations of the LEAS such as the creation of a digital version in addition to further advances in the theory itself. This review aims to provide a comprehensive summary of the evolving theoretical background, measurement methods, and empirical findings with the LEAS. The LEAS is a reliable and valid measure of emotional awareness. Evidence suggests that emotional awareness facilitates better emotion self-regulation, better ability to navigate complex social situations and enjoy relationships, and better physical and mental health. This is a relatively new but promising area of research in the domain of socio-emotional skills. The paper concludes with some recommendations for future research.

## 1. Introduction

Consider the following dilemma. A female consultant in her late 50 s has been working for a demanding female boss for about five years. For the past several months, she has been working remotely because of the COVID-19 pandemic that began in 2020 (ongoing at the time of this writing). The consultant feels that she is underpaid because she is loyal, thorough, accurate and creative in solving problems, and she resents the periodic salary raises she receives because she believes they are too small. The boss states that she is paying her at the top of the pay scale for her job category, which is technically correct. Recently, the boss has started communicating to the consultant in an unfriendly tone that other co-workers notice as distinctly different from how she treats others in the office. The boss has also been complaining directly to her about the consultant’s recent as well as past mistakes, and the consultant is convinced the boss wants her to quit. Privately, the consultant feels that this is very unfair because the alleged mistakes were not her fault. She feels furious and would like to quit, but she needs the money she is earning, however little it is, and finding a new job in the midst of the COVID-19 pandemic is quite difficult. What is she to do?

A healthy approach might consist of figuring out how she feels, why she feels the way she does, and what her feelings tell her about what she needs in the situation. Based on this, she then needs to consider various ways of approaching the situation and then anticipate what the boss’s likely response to each approach will be. Based on her knowledge of the boss, what is the best way for the consultant to proceed? Upon reflection, she realizes that it is the feeling of being mistreated that bothers her the most. She decides that she will speak to the boss directly and explain that she feels that she is being treated both unfairly and in an unfriendly manner, and that she would like to be treated like everyone else. She decides to set aside her resentment regarding her inadequate salary and postpone that conversation for a later date.

This is an example of an everyday circumstance in which socio-emotional skills are called for. Socio-emotional skills may be defined as processing emotional information for the purpose of promoting interpersonal effectiveness. This scenario illustrates how foundational one specific socio-emotional skill—emotional awareness—can be for effective interpersonal behavior in specific contexts. In order to navigate this situation effectively, the consultant must identify her own emotions, interpret what the emotions mean to her, imagine various actions she might take as a result, anticipate what the boss’s emotional responses would be to each option and how she would feel in response, and then decide accordingly on a course of action. All of this involves consciously identifying and examining her own emotions, consciously and differentially imagining how she and the boss would feel in these various iterations, holding these different outcomes in mind in working memory, and then determining what course of action will be most satisfying to her, all things considered. 

In the sections below, we will review a theoretical model of emotional awareness that explains the origin of this ability and its variability across people from psychological, neurobiological, and evolutionary perspectives. Next, we will describe a scale to measure this ability called the Levels of Emotional Awareness Scale (LEAS) and the empirical findings with it in healthy volunteers and clinical contexts. This will allow us to distinguish emotional awareness for self vs. others, and distinguish these from related constructs, such as emotion recognition ability and interpersonal accuracy. In light of this review, we will conclude with a more detailed discussion of the research opportunities that lie ahead in the foreseeable future in this domain of socio-emotional skills. Our aim is to provide the first comprehensive synthesis to date of theory and research on emotional awareness, particularly with regard to emotional awareness as a socio-emotional skill, and to guide future research by highlighting aspects of the theory that remain to be tested.

## 2. A Cognitive-Developmental Theory of Emotional Awareness

### 2.1. The Relation between Emotion and Emotional Awareness

To understand emotional awareness, one must first consider what emotion is. One commonly held view is that moment-to-moment changes in emotional experience correspond to continual and automatic (i.e., without intention or effort) adjustments in physiological, cognitive, and motivational states, based on continual (implicit or explicit) evaluations of the extent to which needs, goals, and values are being met or not met in interaction with the environment. These continual adjustments serve a vital function in human life by supporting behaviors that, in the right contexts, can be adaptive in promoting survival and reproduction (Fridja 1986; Levenson 1994). For example, elevated levels of unpleasant arousal and avoidance motivation, often associated with the experience of fear, can serve to prevent harm in the presence of threat; or the high-arousal, unpleasant approach motivations often associated with the experience of anger can stop unwanted actions by others. Automatic changes in gesture and facial expression in such contexts can also convey information to others, serving a regulatory role in relationships. When each of these aspects of emotional experience are engaged in the right contexts, they can aid in addressing the events in the environment that brought them about (Darwin and Prodger 1998). If events in the environment lead to pleasant experiences, this tends to engage approach motivations; if events instead cause pain or distress, they tend to engage avoidance motivations (Thorndike’s Law of Effect) (Thorndike 1927).

Unlike the automatic physiological and motivational processes just described, conscious awareness of emotion may offer an additional element of adaptive control. Awareness of one’s own emotions involves attending to and reflecting upon one’s automatically generated bodily experiences. Attention and reflection permit extraction of information inherent in an emotional response that helps a person infer what the interaction meant to them and what they need in that situation (Greenberg [2010] 2016). This links directly to the conditions that elicited the response in the first place. Awareness also makes it possible to do something with the information. For example, one can incorporate the information into conscious decision making or change one’s behavior in accordance with a new understanding of what one needs in that situation.

It may not always be appropriate to express automatic affective responses through facial expressions, gestures, or behaviors, depending upon one’s social circumstances (Gross 1999). The case vignette described above is a good illustration of how the unvarnished behavioral expression of an emotion, such as feeling “furious,” might possibly be satisfying in the short term but would likely be disadvantageous in the long term. Awareness of emotion provides a mechanism for internally simulating social interactions and then regulating automatic emotional responses in order to optimize adaptation in both the short and long term. Importantly, being aware of one’s own emotions is also a prerequisite for using that information for the purpose of voluntary emotion regulation in both individual or social contexts (Subic-Wrana et al. 2014). As such, the capacity to be aware of one’s own emotions plays a vital role in the ability to function both as an individual striving for self-actualization and as a social being striving for harmonious social relationships and intimacy (Blatt 2008). 

Success in many such social situations also requires (or at least greatly benefits from) awareness of the emotions of others. Internal simulation and prediction about how others will feel in response to different actions (as exemplified in the case vignette above) will likely be less accurate if awareness of others’ emotions is low. This will depend on related abilities, such as emotion recognition, but only in part. For example, previous studies have consistently shown significant positive relationships between emotion recognition and emotional awareness (Lane et al. 1996; Lane et al. 2000b), and even demonstrated that sex differences in emotion recognition ability are mediated by differences in emotional awareness (Smith et al. 2021a; Wright et al. 2017). However, these relationships are of small effect size—reflecting the fact that awareness of others’ emotions requires more than just recognition ability. Namely, it also requires the ability to internally represent, maintain, and manipulate information about others’ emotions, the ability to predict how their emotions depend on particular events, the ability to differentiate others’ emotions from one’s own (e.g., just because I enjoy doing an activity doesn’t mean another person will also be happy doing that same activity), and the ability to conceptualize others’ emotions in a granular, context-sensitive manner (e.g., the same facial expression can indicate different internal states in different contexts (Aviezer et al. 2008; Barrett et al. 2011)). This plausibly draws on the use of background knowledge to facilitate interpersonal accuracy (i.e., accurately inferring others’ states and traits; Schmid Mast and Hall 2018). However, as we will see, unlike standard measures of recognition and inferential accuracy, current measures of emotional awareness for others do not focus on accuracy norms. Instead, they focus only on the level of granularity and self-other differentiation when individuals describe how they believe others would feel in hypothetical situations (i.e., just on the structure and sophistication/complexity of the emotion concepts invoked and how they are used). Thus, emotional awareness for others can be identified with the ability to simulate the granular, differentiated emotions of others that would be evoked in specific situations and to use that information in a goal-directed manner—allowing the effective management of others’ emotions, as also found in a recent study (Smith et al. 2021a).

### 2.2. A Theory of Emotional Awareness as a Cognitive Skill

In 1987, Lane and Schwartz proposed that an individual’s ability to recognize and describe emotion in oneself and others (i.e., emotional awareness) is a cognitive skill that undergoes a developmental process similar to that which Piaget described for the development of other cognitive capacities (Lane and Schwartz 1987; Piaget 1937). Just as Piaget described the transformation from bodily-based, enactive representations to abstract, conceptual representations as a general framework for understanding cognitive development, Lane and Schwartz were the first to apply this framework to emotion. A fundamental tenet of this model is that individual differences in emotional awareness reflect variations in the degree of differentiation and integration of the schemata (implicit programs or sets of rules) used to process emotional information, whether that information comes from the external world or the internal world through introspection. Emotional awareness is considered to be a separate line of cognitive development that may proceed somewhat independently from other psychological domains (Lane and Schwartz 1987). A more detailed discussion of the model from a Piagetian perspective was published 15 years after the first paper (Lane and Pollerman 2002). 

This theoretical perspective is consistent with more recent perspectives on both cognitive development and emotion. Karmiloff-Smith (1992) argued that cognitive development consists of the process of “representational redescription,” whereby procedural representations are transformed by (i.e., mapped to) abstract conceptual representations, thus providing two different ways of knowing—with the latter being more complex, flexible, and adaptive. This dovetails with Barrett’s constructivist theory of emotion, which holds that specific emotions result from conceptualizing “basic affect,” where the latter consists of relatively undifferentiated valenced bodily states (Barrett 2017). As such, the fundamental driver of increasing emotional awareness is hypothesized to be the process of conceptualizing emotion-related bodily experiences, which most commonly involves putting emotions into words. The more one does this, the more Werner’s “orthogenetic principle” applies, which states that “wherever development occurs it proceeds from a state of relative globality and lack of differentiation to a state of increasing differentiation, articulation, and hierarchic integration” (Werner 1957). 

The model posits five “levels of emotional awareness” that share the structural characteristics of Piaget’s stages of cognitive development and constitute a continuum ranging from global, undifferentiated states to more differentiated and integrated states (Piaget 1937). The levels of emotional awareness in ascending order are (1) awareness of physical sensations, (2) action tendencies, (3) single emotions, (4) blends of emotions (i.e., feeling multiple emotions at once), and (5) blends of blends of emotional experience. This highest level encompasses the ability to distinguish blends of emotions in self vs. others (e.g., “I would feel sad and ashamed, while the other person would feel happy and excited”), which is the basis of how it is scored in the Levels of Emotional Awareness Scale (see below for details). However, with regard to Emotional Awareness theory, this level can also refer to the ability to imagine different blends of emotions that might be experienced by the same individual (self or other) under different possible scenarios (e.g., when considering the future outcomes of different possible actions).

Unlike stages of cognitive development, which were thought to emerge sequentially in development, these levels constitute modes of functional organization that can shift in either direction at any time. The feelings associated with a given emotional response can include the contents of each of these levels, up to and including the highest level attained. From this perspective, the different levels are associated with momentary states. Each level is also associated with specific characteristics, including the subjective quality of emotional experience, the degree of differentiation of emotion, the ability to describe emotion, and the degree of self-other differentiation. Thus, each level has a coherence and stability of its own, and individuals tend to function at or near a consistent level (Versluis et al. 2018). See Table 1 for a description of the five levels. 

**Figure 1:**
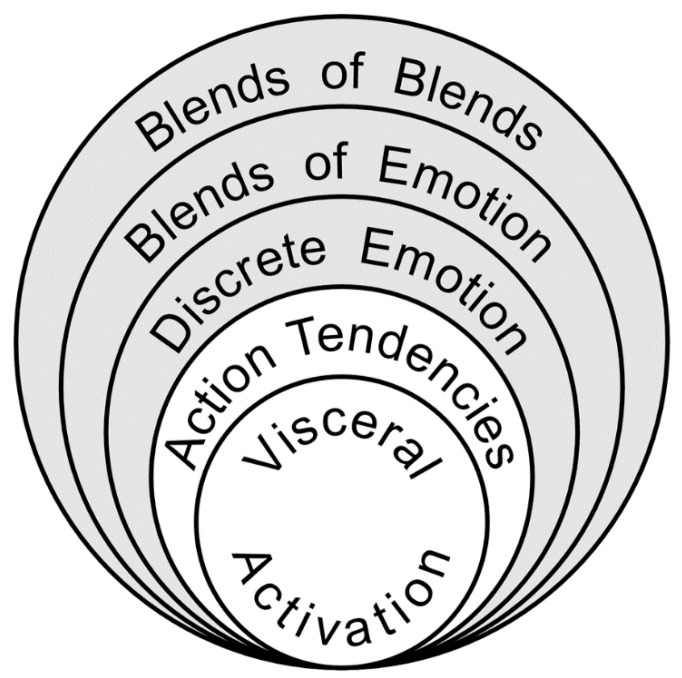
Graphical depiction of Levels of Emotional Awareness as a nested hierarchy.

The levels are aligned in a nested hierarchy as illustrated in Figure 1 such that functioning at each level adds to and modifies the functioning of previous levels (but does not eliminate them). For example, blends of emotion (Level 4 experiences), compared to action tendencies (Level 2 experiences), are associated with more differentiated representations of somatic sensations (Level 1) (Lane et al. 2011). Similarly, higher levels of emotional awareness are associated with greater heart rate variability at rest (a measure of physiological differentiation over time in cardiac vagal control, a visceromotor [level 1] function) (Verkuil et al. 2016). This influence of higher levels on lower levels is an indicator of progressively increasing integration. The five levels, therefore, describe the cognitive organization or complexity of emotional experience as manifested in the description of one’s experiences, not simply one’s thoughts about or appraisal of one’s emotions. Generating such differentiated descriptions plausibly depends on attending to one’s emotions, reflecting on their meaning, and having the repertoire of emotion concepts needed for fine-grained understanding.

### 2.3. Development of Emotional Awareness

Because emotional awareness pertains to acquiring granular emotion concepts and the automatic tendency to attend to (and value) emotional information, it follows that emotional awareness is learned (although innate differences could facilitate or hinder this learning process, e.g., personality traits associated with affective volatility or openness to experience; Lane and Schwartz 1987; Smith et al. 2019b). As with any other kind of learning, acquiring emotion concepts and adaptive attentional habits therefore requires signals to learn from. In the case of emotion concepts, this plausibly involves being exposed to a range of emotional responses in others, experiencing a range of emotional responses in oneself, having attention directed toward these responses, and being exposed to emotion labels to aid in categorization (among others). In the case of attention to emotion, this plausibly involves reinforcement learning processes in which attending to emotions in self and others is repeatedly followed by positive feedback or other beneficial outcomes. In the absence of such signals, such as with early abuse/neglect, one may not develop emotional awareness and only achieve a low level of awareness in adulthood (although, as described below, interventions have been successful at improving emotional awareness in adults—by providing the teaching signals that may have been absent in childhood; Burger et al. 2016; Montag et al. 2014; Neumann et al. 2017; Radice-Neumann et al. 2009; Smith et al. 2021b; Subic-Wrana et al. 2005). If socio-emotional signals were selectively lacking in childhood, it is possible that other aspects of cognitive development could still proceed normally—allowing for divergence between emotional awareness levels and other cognitive skills acquired in development.

A foundational starting point for understanding these learning processes is that emotions are interpersonally regulated in childhood (Fonagy et al. 2018). In fact, the child may learn about its own emotional responses largely through the attunement, empathy, mirroring, and responsiveness of the caretaker (typically the mother) (Beebe and Lachmann 2013). Beginning at birth, infants have a very limited range of emotional expressions. For example, what appears to be the same behavior of crying can occur for a wide variety of reasons, such as being hungry, thirsty, tired, cold, having a soiled diaper, being in pain, or being surprised. It is the caretaker’s job to figure out what is going on and provide the needed remedy. In so doing, by virtue of the caretaker figuring out what the child’s bodily state and emotional expressions mean, and by inducing relief from distress through targeted action (among other kinds of contingent responsiveness), the child can gradually begin to learn that its bodily states have meaning (Fotopoulou and Tsakiris 2017; Gergely and Watson 1996; Lane et al. 2018). In the absence of such responsiveness, the young child will have automatic emotional responses but will have limited means of coming to an understanding of what they are or what they mean. Experiences in orphanages (Colvert et al. 2008) or foster care (Pears and Fisher 2005), where physical but not emotional needs are met, have demonstrated that such deprivation is associated with impairments in self-regulation, socio-emotional cognition, and, if persistent, even death (Croughs 1971). 

As the child grows older and develops a broader emotion repertoire (e.g., see Widen and Russell 2008), clues to what the child is feeling might include a wider variety of facial expressions, gestures, and other actions that constitute the observable nonverbal expression of emotions. The latter, in combination with the social or environmental context in which the responses arise, makes it possible for an observer to guess what the child may be feeling emotionally. Mirroring of a child’s emotions by its caretaker provides visual and vocal information congruent with interoceptive sensations that enables conceptualization of internal experience to begin (Beebe and Lachmann 2013). Once language develops, words become an essential tool in advancing one’s conceptual knowledge of what one is feeling in interaction with an attuned other. A leading theory of emotion (already touched upon above) argues that differentiated emotional experiences are constructions that are created by linking bodily sensations with concepts, the latter of which is greatly facilitated by language (Barrett 2017). Current theory suggests that the more emotional feelings are responded to nonverbally and then discussed and dissected in one’s daily routine, the more advanced emotional awareness can potentially be. This might include finding appropriate verbal labels for feelings; understanding what the circumstances were that triggered those feelings; appreciating how emotions feel in one’s body; and recognizing that felt emotions are associated with bodily expressions that may be visible to others (and that one may or may not want others to see). Strategies for regulating and expressing emotions also appear to be learned, at least in part, through a combination of observation (modeling) and interactions with others (Beebe and Lachmann 2013; Kleiman-Weiner et al. 2020). Over time, the child would then begin to develop a repertoire of emotion concepts and a corresponding vocabulary that make it possible to understand and describe one’s own feelings, the feelings of others, what the feelings mean, and what to do about them (Barrett et al. 2001). As a result, the capacity for emotional awareness grows alongside the capacity for emotion self-regulation. The theory of levels of emotional awareness is consistent with this work in predicting that awareness should increase throughout development (consistent with positive associations with age and emotional awareness scores observed in children and adolescents; (Agnoli et al. 2019; Mancini et al. 2013; Veirman et al. 2016)), and that the highest level attained will depend on the quality of social interactions with early caretakers and peers, as well as on subsequent social interactions during later childhood and adolescents (e.g., school settings).

### 2.4. An Evolutionary Perspective on Emotional Awareness

In a recent paper, we proposed that the human species is unique in its capacity for emotional awareness—that is, humans have a unique ability to attend to and reflect upon their automatic emotional responses and decide how to manage or express them before taking actions observable to others (Smith et al. 2020b). We proposed that this capacity arose from a combination of domain-general (i.e., general cognitive) and domain-specific (i.e., specialized socio-emotional) mechanisms. Here domain-general simply refers to mechanisms that have access to, and can operate on, a wide variety of information sources; domain-specific refers to mechanisms that only operate on subdomains of available information (e.g., interoceptive signals, facial dynamics, etc.). Regarding domain-general mechanisms, we argued that the disproportionate cortical expansion during human evolution reflects additional hierarchical levels of processing, allowing representation of multimodal regularities over longer timescales—affording abstract concept learning, internal simulation of distal future outcomes, and expanded working memory capacity. This allows for the ability to simulate emotions, learn emotion concepts, and manipulate them in working memory when deciding how to act, as in the opening vignette above. We then argued that the phylogenetically older automatic response generating functions, which in many ways are shared with other species and have been described as survival circuits (LeDoux 2012), interact with the domain-general functions. In part, this occurs indirectly through the visceral responses generated by these survival circuits, which are then perceived (e.g., through interoception) and can be conceptualized as related to emotions. These interactions also allow top-down, context- and goal-dependent regulation of survival circuit activity and response generation. As such, this proposal is highly consistent with the formulation described above, in which general principles of cognitive development can be shown to apply to specific content areas such as emotion (i.e., if/when one learns to effectively apply domain-general cognitive processes to socio-emotional signals).

A key point in this model is that we viewed these changes in the evolution of the human brain as simply creating the capacity for emotional awareness. We proposed that development of higher levels of emotional awareness in a given person then further depends on a cognitive/behavioral calibration process associated with the construct of life history strategy (LHS) in evolutionary psychology (Figueredo et al. 2005). Life history theory describes how, for any species, its rate of reproduction varies as a function of whether the local conditions are safe and secure or harsh and unpredictable. In the latter case, the best strategy for perpetuating the species, called a *fast life history strategy*, is to have many offspring and invest relatively little in each—and to focus on short-term over long-term rewards (i.e., because long-term outcomes are unpredictable and mortality is high). By contrast, in the case of *slow life history strategy*, more resources, both material and psychological, can be invested in any given child to ensure their survival and functional viability—and effortful cognitive processes focused on achieving long-term goals is adaptive (i.e., because long-term outcomes are predictable and mortality is low). Crucially, while LHS varies between species, it is also thought to be further calibrated during early development within species, such that, in humans, harsh and unpredictable early environments (e.g., childhood abuse/neglect, neighborhood violence) promote development of fast LHS traits—such as short-term focus, higher risk-taking, multiple shallow sexual relationships, and less investment in social relationships generally—which would be adaptive in such environments (i.e., where relationships and long-term choice outcomes truly are unpredictable). This pattern of reduced engagement of reflective (effortful, long-term focused) cognition and less investment in relationships would be expected to reduce opportunities to learn about and reflect upon emotions.

Based on these considerations, we proposed that this framework applies to emotional awareness, in that the ability of a child to learn about and understand emotions is facilitated within a slow life history setting that permits attuned attention and empathy from a caring, dependable adult (most typically a parent) who can recognize the child’s needs and respond to them in a consistent and effective way. This is a luxury of sorts, which is possible once other basic needs (e.g., food, shelter, clothing, etc.) are met. In so doing, the child can learn that its bodily sensations (including those associated with emotional responses) have meaning, that distress is a temporary state, and that effective interventions are possible. In a fast life history context, neglect or abuse may be common and the ability to learn what internal emotional signals mean may often be impaired, (i.e., parental interactions facilitating emotion learning may be absent and the outcomes of emotional responses may be inconsistent).

Since we put forward this hypothesized relationship between emotional awareness and LHS, two studies have tested this hypothesis and found significant correlations showing that higher emotional awareness is associated with slower LHS and greater early adversity (Smith et al. 2021a, 2021c). The components of LHS most related to emotional awareness in these studies were general differences in cognitive reflectiveness and supportiveness of parents in childhood. However, future research is needed using a wider range of measures. Specifically, while the aforementioned studies used psychometric measures of LHS (Figueredo et al. 2017; Manson et al. 2020), it remains to be shown whether emotional awareness is associated with other commonly used biometric/demographic measures (e.g., neighborhood stress and socioeconomic status, pubertal timing, timing of first sexual behavior and reproduction, short- vs. long-term sexual relationships, delay discounting, among others; see (Chua et al. 2020; Mededovic 2019, 2020)).

An important implication of this formulation is that a child’s emotional awareness is both calibrated by, and adapted to, the social environment in which the child finds itself. This process was likely highly adaptive for the environmental niche in which the modern human brain evolved, as it promoted social cohesiveness within the limited social circles of the pre-civilization hunter–gatherer way of life. It also highlights how acquisition of higher levels of emotional awareness is not necessarily better in an absolute sense, but is determined by a combination of individual and social considerations in an environmental context. For example, the focus on short-term rather than long-term gains in a fast life history strategy, and associated minimal investment/trust in long-term relationships, may actually be the most adaptive in such contexts (i.e., where long-term outcomes and relationship stability are not predictable). This is because the psychological investment in acquiring and applying emotional awareness is only beneficial in cases where patterns of emotional responses are sufficiently stable, and relationships are sufficiently long-lasting, to allow this awareness to usefully guide decision-making. In contexts where emotional response patterns are instead volatile, and where relationships change quickly, emotional awareness may not offer sufficient predictive utility to be worth the investment to acquire it. 

Crucially, despite adaptiveness in congruent contexts, problems can arise later in life if there is a mismatch between the strategy learned in childhood and the social circumstances encountered in adulthood (e.g., if a child who acquired a fast LHS moves into a slow LHS environment, such as a professional work setting). Consider, for example, how the consultant in the case vignette above would fare if she had acquired a fast LHS—and only short-term gains were the principle guiding behavioral decision-making when interacting with the boss who was perceived as unfair. Expressing her fury at being treated unfairly might be satisfying in the short run, but this would be unlikely to serve her best interests in the long run. Unlike the fast life history environment in childhood, acquisition and use of emotional awareness is worth the cognitive investment in such slow life history settings.

These considerations highlight ways in which difficult dilemmas can arise. For example, if LHS mismatches of this kind are experienced later in life within occupational settings—where prevailing value systems differ significantly from those learned earlier in life—should one change jobs or learn to adapt? On another hand, consider a scenario in which a person marries someone with a similar fast life history background, but becomes unhappy after exposure to the trade-offs and advantages of a slow life history strategy in their adult environment (e.g., in the media, or from real-life experiences in the wider social world). This could motivate adaptation and development of increased emotional awareness (consistent with a slower life history strategy). However, doing so could create conflicts and difficulties in adjustment to the marital relationship, unless the marital partner is also willing to engage in a similar learning process. For example, the less reflective, impulsive emotional behavior associated with fast LHS might no longer appear reasonable to a partner who has become more empathetic and reflective about emotions and has come to expect that their partner should do the same. Another possibility is that conflict could arise due to resulting differences of opinion regarding correct parenting strategies (e.g., differing levels of attention and investment in each child’s wants and needs).

Consistent with these considerations, there is also related work suggesting that higher emotional awareness may not always be better in all contexts. For example, one study found that higher emotional awareness was associated with greater levels of anxiety in generalized anxiety disorder (Novick-Kline et al. 2005), suggesting a potential drawback of continued attention toward one’s own emotions. Another study found greater affective priming effects in those with high emotional awareness (Suslow et al. 2001), suggesting an increased automatic sensitivity to emotional cues. While this could be helpful in some contexts, it might also interfere with goal-directed cognition/behavior in others. Related work has similarly discussed potential downsides of accurate perception of others’ emotions (for a review, see Schlegel 2020). For example, while romantic relationships often benefit from high emotion recognition ability, this ability could cause problems in relationship-threatening contexts (e.g., where relationship stability might be improved by not being fully aware of the mixed feelings of one’s partner). Further, individuals with higher emotion recognition ability do not indicate greater levels of health, happiness, or life satisfaction that one might expect (Schlegel 2020). More research is therefore needed to understand when high levels of emotional awareness and related skills are and are not helpful to personal well-being.

### 2.5. A Computational Neuroscience Perspective on Emotional Awareness

The evolutionary theory just described incorporated fundamental principles of computational neuroscience. One of the leading themes in recent computational neuroscience research is predictive processing (Hohwy 2014). The predictive processing framework posits that perceptions of the external environment are not based solely on sensory input. Instead, the brain is thought to continuously make predictions about new sensory input based on current beliefs (i.e., based on its internal model of the world). The primary role of sensory input is then to correct those beliefs when predictions are inaccurate (i.e., perception involves finding the minimal change in beliefs that will minimize the difference—or prediction error—between predicted and observed sensory input). Because the body is external to the brain (i.e., just as is the rest of the environment), perceptions of the bodily states associated with emotions are also influenced by predictions—and these predictions depend on the concepts that have been learned and incorporated in the brain’s internal model of the world (Seth 2013; Seth and Friston 2016). The domain-general brain mechanisms that were expanded during human evolution made it possible to encode, recognize, and use regularities over longer time scales—expanding the capacity to consider long-term vs. short-term goals. Higher levels of emotional awareness can be understood as a consequence of the complexity of the prior beliefs used (and associated predictions made) to interpret bodily sensations in various social contexts—where this set of predictions is derived from one’s social learning history. These evolutionary and computational perspectives therefore dovetail nicely with Piagetian and post-Piagetian perspectives on the cognitive-developmental contributions to emotional awareness. 

Concrete demonstrations of the computational account of emotional awareness described above have also been provided in simulation work demonstrating at least seven different computational mechanisms capable of producing a low emotional awareness phenotype (Smith et al. 2019b, 2019c). For example, someone with low emotional awareness due to an underdeveloped repertoire of emotion concepts, often associated with emotional neglect and/or fast life history strategy, may interpret bodily affective states in a coarse-grained and undifferentiated way (e.g., thinking in terms of pleasant vs. unpleasant, instead of distinguishing between different pleasant/unpleasant emotions). As another example, if an individual develops overly precise/rigid prior beliefs about expected emotional or bodily states in a given situation, afferent sensory signals may become unable to effectively update those beliefs (e.g., an individual might inappropriately interpret unpleasant, emotion-related sensations as signs of sickness or other somatic threat). This would in turn drive peripheral physiology in a way consistent with the metabolic demands entailed by those inaccurate interpretations (e.g., sustained elevated arousal to meet the expected threat). One leading computational account that uses predictive processing as a means of controlling visceromotor regulation in this manner is called active inference (Pezzulo et al. 2015). The failure to update beliefs/predictions based on bodily feedback, as in low emotional awareness, can lead to what might be considered inaccurate or inappropriate physiological and behavioral responses based on the value system of one’s current social circumstances. Recent theorizing suggests that this is a useful way of understanding the contribution of impaired emotion processing to the pathophysiological basis of systemic medical disorders associated with persistent, undifferentiated arousal (Peters et al. 2017), as well as functional somatic syndromes (Henningsen et al. 2018) and functional neurological disorders (Pick et al. 2019). Fortunately, evidence is now available demonstrating that psychoeducational as well as psychotherapy modalities can promote this type of emotional learning in adulthood and improve emotional awareness (Burger et al. 2016; Killgore et al. 2020; Montag et al. 2014; Neumann et al. 2017; Radice-Neumann et al. 2009; Subic-Wrana et al. 2005). Given the complexities and social challenges associated with such learning that were noted above, however, it is not to be undertaken lightly. 

With the realization that the brain is a predictive organ (Hohwy 2014), there has also been a reappraisal of what emotions are and how they function to promote adaptation of the organism and the organism’s body as it interacts with the external world (Barrett 2017). This new perspective addresses the dual challenge of predicting and adapting (both viscerally and behaviorally) to what is likely to happen next in the external world (called allostasis) (Sterling 2012), while also maintaining the kind of stability within the internal milieu that is necessary to sustain life (called homeostasis) (Cannon 1929; Modell et al. 2015; Petzschner et al. 2017). Homeostasis is a self-regulating process that works by negative feedback to return to a set point after a deviation has occurred. For example, when blood glucose increases, insulin is released to bring glucose levels back to baseline. Whereas homeostasis is reactive, this must be reconciled with the reality that the brain, which must navigate and coordinate both the internal and external worlds, is predictive. In fact, relying on negative feedback alone is inefficient (Sterling 2012). Allostasis, on the other hand, involves the predictive regulation of the internal milieu by anticipating what is needed to meet the challenges in the near future posed by the external world (e.g., increasing insulin levels in advance due to expected increases in blood glucose; from computational simulations, see (Stephan et al. 2016; Tschantz et al. 2021)).

Evidence is accumulating to suggest that emotion works allostatically in the service of homeostasis (Barrett 2017). Here it is useful to consider that automatic emotional responses likely evolved from the valenced states that govern basic bodily processes (Damasio and Carvalho 2013). Basic homeostatic functions such as hunger and thirst include subjective experiences that are valenced in the sense of being associated with a pleasant or unpleasant feeling tone in the body. When a physiological need that promotes survival is satisfied, it feels good; if a physiological need is compromised or unmet, it feels bad. Behaviors associated with good feelings tend to continue (until the need is met), whereas behaviors associated with bad feelings tend to be avoided (until the danger or threat has subsided). Whereas homeostatic emotions pertain to bodily states essential for survival (independent of the cause of the deviation from homeostasis), emotions in a more general sense extend this capacity for valenced experiential responses to interactions with the environment, including the social environment, that are perceived exteroceptively and are much more numerous and differentiated than the limited number of homeostatic feelings associated with basic bodily functions (i.e., which are perceived through interoception alone). 

The feelings associated with emotional responses are thought to reflect hierarchical sets of representations that construct a model of particular aspects of the internal/external world. This includes representations of the basic somatomotor and visceromotor responses that anticipate what is needed to interact with the environment, as well as concept-level emotion representations of the meaning of this interaction (Barrett 2017). As such, approach and avoidance behaviors and the physiological changes that support them are the foundation for the subjective feeling that emerges, consistent with a Piagetian perspective on emotion. Emotion in this basic sense is implemented allostatically and serves a fundamentally life-preserving homeostatic function. Although there is no way of knowing for sure, comparative neuroanatomy suggests that the phenomenal feelings associated with such emotional responses likely occur in other animals as well (Damasio and Carvalho 2013; Panksepp et al. 2017).

Unlike other animals, however, human beings possess the unique ability to be aware of their own emotional responses, i.e., to conceptualize what they are feeling (Smith et al. 2020b). This awareness may ultimately serve an allostatic function by making it possible to identify the personal need inherent in the emotional response and to use conscious cognition and decision making to predict what actions will be necessary to meet that need (i.e., keeping the body in homeostatic ranges over the long run). According to Watson and Greenberg (2017), mindful awareness of emotion consists of first attending to the bodily responses associated with emotions, conceptualizing or symbolizing those bodily sensations as emotions, and ensuring that the words selected fit the bodily feelings experienced as well as the context that elicited them. It then involves accepting that one is having those feelings, having a sense of agency or ownership over them (instead of being controlled by them), and then regulating them and differentiating between the various emotions that may constitute the entire subjective response to a given situation. In contrast to emotional responses that put one in a given state, awareness enables agency or ownership, and thus an ability to influence that state in an intentional manner that optimizes future adaptation. For example, awareness of one’s own emotional responses may help enable conscious, intentional self-regulation of these responses, such as suppression or reappraisal (Gross 1999), which facilitates individual and social goal attainment, depending upon the circumstances (e.g., as suggested by Subic-Wrana et al. 2014). This process is also down-regulatory in several senses. Self-awareness has been found to engage brain regions (e.g., within the default mode network) that promote vagal tone (Thayer et al. 2012); this may account for the positive correlation between LEAS and heart rate variability at rest (Verkuil et al. 2016). Although reflecting upon one’s own experience temporarily increases the intensity of the experience (Thompson et al. 2011), putting emotions into words has an automatic inhibitory effect on emotional responses (Lieberman et al. 2007)—thus facilitating the return to baseline and the ability to respond to any subsequent changes in the environment. This inhibitory function does not squelch emotional responding entirely, but rather promotes differentiation and the appreciation of layers of experience (Thayer and Lane 2000), such as feeling angry and sad about the same situation. It is important to emphasize that higher-level skill is not universally observed; instead, skill level varies across individuals as a function of life history strategy and other variables (as described earlier). 

The above considerations illustrate how emotional awareness may therefore permit additional socio-emotional regulatory functions that can be broadly conceptualized in terms of allostatic processes. Namely, by knowing what external social circumstances have triggered (or are expected to trigger) an emotion, one can predict and take the appropriate action to alleviate (or prevent) that trigger, particularly when the emotion is aversive. For example, occupational or family circumstances may be ongoing and constitute persistent sources of distress. As in the opening vignette in this paper, recognizing the link between one’s own emotional responses and the meaning of the circumstances triggering them, consideration of the feelings of others involved, anticipating how self and others would feel depending upon how one responds (etc.) all require the application of emotional awareness in anticipatory interpersonal problem-solving. Addressing the trigger directly to alter it, or accepting the situation so that one is no longer so disturbed by it, constitutes this added capacity (also discussed elsewhere in terms of situation- and response-focused emotion regulation strategies; (Gross 1998a)). This would be quite difficult without higher levels of emotional awareness, which facilitates the ability to maintain and manipulate differentiated responses of self and others in working memory until a suitable response is arrived at and implemented (Lane et al. 2020b; Smith et al. 2017b).

In summary, from this perspective, awareness of one’s own emotional states can allow one to capitalize on the brain’s domain-general functions to extend the ability to anticipate and adapt to challenges in the external world by incorporating longer timescales, which importantly includes the social world, above and beyond what is provided by automatic affective responses. Thus, awareness enables a more detailed and intentional use of information inherent in basic affective responses—with regard to the meaning of the interaction for both the self and others, as well as the meaning of the context in which the feelings arose. As such, it facilitates decision making that can enable the achievement of longer-term goals than those addressed by automatic emotional responses.

### 2.6. Relation between Emotional Awareness and Other Constructs in Research on Socio-Emotional Skills

There now exists a large body of research on inter-related socio-emotional skills, many of which fall under the broad umbrella construct of emotional intelligence (EI). It is important to consider how these other skills may relate to emotional awareness. The most widely-used performance measures of EI—associated with models that conceptualize EI as a set of abilities—are designed to test the capacity to recognize emotions in others, understand emotions and how they change over time (and combine with other emotions), use emotional information in thought, and regulate one’s own emotions as well as those of others (see the Mayer-Salovey-Caruso Emotional Intelligence Test [MSCEIT]; Mayer et al. 2003). Self-focused emotional awareness has received less direct attention in these performance-based measures; but several of the skills measured in EI tests have clear relationships to other-focused emotional awareness, and some may indirectly depend on (or be facilitated by) self-focused emotional awareness as well. The most plausible overlap is with subscales in EI tests—that measure emotion understanding—as a link between emotion perception and emotion regulation. As is the case with emotional awareness, if one does not have a thorough conceptual understanding of the emotions perceived in others, and their dependence on situational events, this would be expected to hinder the ability to use that emotional information to effectively guide social interaction. This is also discussed in related models of affective social competence, which focus on awareness as an important mediator between receiving and sending affective signals in an adaptive manner (Halberstadt et al. 2001). 

Consistent with these considerations, a few previous studies in both adults (Barchard and Picker 2018) and children (Barchard et al. 2010a; also see Bajgar et al. 2005) have reported significant but small effect size, relationships between emotional awareness (LEAS scores) and performance-based tests of EI, as well as related measures of emotion comprehension and social skills. In adults, these studies have found that emotional awareness scores show significant positive correlations with the “perceiving”, “understanding”, and “managing” subscales of the MSCEIT (*r*s = 0.20 to 0.35), as well as with tests of understanding emotion metaphors (*r* = 0.33; Barchard et al. 2013) and multiple aspects of social intelligence more broadly (*r*s = 0.21 to 0.31; O’Sullivan and Guilford 1976). In children, these studies have found that emotional awareness scores are positively associated with tests of both emotion recognition (Fine et al. 2003) and emotion comprehension (Cermele et al. 1995; Garner et al. 1994). One further neuroimaging study in adults (Smith et al. 2017b) also reported a positive correlation between emotional awareness scores and total scores on the MSCEIT in adults, with a similar magnitude to the other results mentioned above (*r* = 0.26), but this study did not assess subscale scores and the small sample size (N = 26) was underpowered to detect small effect sizes. This same study found a similar magnitude positive correlation (*r* = 0.23) with total scores on a self-report measures of EI (the TEIQue; Mikolajczak et al. 2007)—based on models that instead conceptualize EI in terms of self-perceived traits (Petrides et al. 2016). However, it also did not report on subscales for this measure (and the correlation was not significant given the small sample size). Another study in a large sample of children found a positive but low effect size (*r*s = 0.12 to 0.15), correlation between scores on children’s versions of the LEAS and TEIQue (Agnoli et al. 2019). Subscales on such self-report EI measures typically ask individuals about their self-perceived awareness and understanding of emotions (as well as related subscales regarding empathy), which might be expected to relate to emotional awareness measures. However, one might also expect weaker relationships between self-report and performance-based measures of emotional awareness, as has been observed with self-report and performance-based EI measures (Austin 2004; Petrides 2011; Webb et al. 2013). Self-report measures of alexithymia, which ask individuals similar questions about their understanding of emotions (Parker et al. 2001), have also not been found to correlate strongly with performance-based emotional awareness measures (Maroti et al. 2018). While the studies linking emotional awareness scores to other performance-based emotional skills measures provide some initial insights, they will need to be replicated and extended in future work. Notably, more recent studies have also described novel measures of emotion understanding (Schlegel and Scherer 2018), which would also be predicted to correlate with emotional awareness scores. As noted above, however, the scoring of performance-based measures of emotional intelligence and emotion understanding consists of determining whether respondents provide correct answers, whereas there are no correct answers on the LEAS. Thus, statistically significant but low magnitude correlations would be expected.

Stepping back from specific measures, at the conceptual/theoretical level there is a notable consistency between the levels of emotional awareness framework and more recent models of emotion understanding in both children and adults (Castro et al. 2016; Elfenbein and MacCann 2017; Fiori et al. 2019; Schlegel and Scherer 2018). For example, one recent model of emotion understanding has distinguished between emotion recognition and emotion knowledge (Castro et al. 2016), each of which have shared and unique aspects for self, others one knows, and others one does not know. Separate consideration of emotion knowledge in childhood vs. adulthood also mirrors work on emotional awareness in children vs. adults described below. Recently proposed revisions to the ability model of EI have further proposed specific subdomains regarding emotion understanding and emotion expression (Elfenbein and MacCann 2017; Mayer et al. 2016). These constructs overlap with emotional awareness in that both allow for the ability to predict emotional reactions, identify their causes, form expectations about how they will evolve over time, and/or articulate them in granular ways. In contrast to cognitive reasoning and regulatory components of EI, recent work (Fiori et al. 2019) has also highlighted a separable sensory-motor emotion-information processing component, which they relate to fluid IQ—which could potentially overlap with the bottom-up, perceptual, real-time processing aspects of emotional awareness (e.g., as they relate to linking bodily sensations to emotion concepts).

One last construct with the closest theoretical links to emotional awareness is emotion differentiation (Erbas et al. 2019; Kashdan et al. 2015). Measures of emotional awareness have even previously been discussed as a means of assessing emotion differentiation (Kashdan et al. 2015) because emotional awareness scores are higher when individuals use more differentiated emotional descriptors (e.g., feeling sad/afraid/angry as opposed to just feeling bad/unpleasant; see below for more details). However, other available measures of emotion differentiation take distinct approaches to operationalize and measure differentiation, and few direct tests of the relationship between emotional awareness scores and these other measures have been performed to date (e.g., one study found a positive relationship between emotional awareness scores and greater within-category variance in self-reported emotional experiences; (Smith et al. 2019d)). Empirical studies further assessing relationships between these measures will also be important.

## 3. Research Using the Levels of Emotional Awareness Scale

### 3.1. Description of the Scale

Given the theory that there are individual differences in the differentiation and complexity of emotional experience, and that lower levels of emotional awareness are associated with a focus on somatic sensations, we sought to create an assessment method that would enable us to quantify such individual differences. However, a self-report scale that prespecified the experiences in the item content would defeat the purpose of determining how respondents spontaneously structure their experience. We also considered that, if we sought to identify people who were not aware of their own emotions, an assessment method in which people rated themselves on their own ability to be aware of their emotions would be compromised by the impairment that we sought to measure. We also appreciated the need to determine how such processes applied to the anticipated experience of others as well as oneself. We therefore created a performance measure that consisted of a direct display of the ability being assessed. This measure is therefore quite different from standard methods of assessing emotional experience, which typically specify the emotion terms and ask the respondent to rate the intensity or frequency of experience over a given time period.

The resulting measure—the Levels of Emotional Awareness Scale (LEAS)—asks a person to describe his or her anticipated feelings and those of another person in each of twenty vignettes described in two to four sentences (Lane et al. 1990). The only instruction is that participants are required to use the word “feel” in their answers. Scoring is based on specific structural criteria aimed at determining the degree of differentiation in the use of emotion words (i.e., the degree of specificity in the terms used and the range of emotions described) and the differentiation of self from other. Participants are not informed about the basis of scoring or any other information about the aims of the test. The scoring is guided by a scoring manual (Barchard et al. 2011) and involves little or no inference by raters (see Table 2). Due to the strong reliability and validity data available (see below), the LEAS has been selected for inclusion in the National Institutes of Mental Health Research Domain Criteria Matrix under the following headings: Domain: social processes; Construct: perception and understanding of the self; Subconstruct: self-knowledge (https://www.nimh.nih.gov/research/research-funded-by-nimh/rdoc/units/self-reports/151126.shtml, accessed on 8 August 2021). To date over 180 papers and chapters, many of which are cited in this paper, have been published on the scale or its application (listed at eleastest.net).

The LEAS identifies five levels of emotional awareness, from low to high: (1) bodily sensations (e.g., sleepy), (2) global, undifferentiated emotions or action tendencies (e.g., good, feel like striking out), (3) single emotions (e.g., happy), (4) blends of emotion (e.g., “I would feel sad and disappointed”), and (5) combinations of blends in self and others (e.g., “I would feel sad and angry; the other person would feel happy and relieved”). Each item of the LEAS presents a hypothetical emotion-eliciting social scenario including the participant and another individual. Each word in the written responses is then scored using an extensive glossary (i.e., 0 = nonemotion word, 1 = bodily sensation, 2 = valence/approach-avoidance behavior, 3 = specific emotion word, 4 = two or more specific emotion terms) and a coding scheme is used to construct a single score per scenario. Specifically, each of the self-focused and other-focused sub-items is separately given a score of 0–4 (based on the highest-level emotion term(s) used, independent of text length), and these sub-item scores can separately be summed to provide an overall self-focused and other-focused EA score. The total score for each item represents the higher of the self- and other-focused scores. In addition, for any item where both the self- and other-focused sub-items receive a score of 4, the total item is instead given a score of 5—so long as the self- and other-focused descriptions are differentiable (for details see Lane et al. (1990); go to eleastest.net to acquire the scoring manual). The total score (0–5) for each item is then summed to provide a total measure of emotional awareness—with higher scores indicating greater awareness and differentiation in emotions. 

Internal consistency of the LEAS Total score as measured by Cronbach’s alpha is high for the 20-item version and very good for the 10-item version (see Table 3). Inter-rater reliability is excellent (see Table 4). Intra-rater reliability by an expert rater who scored 16 protocols two years apart was *r* = 0.993. The test–retest reliability at four weeks has been shown to be quite good (Spearman Brown correlation = 0.80) (Torrado et al. 2013). Norms for age, sex, and socioeconomic status have been established (available upon request from the corresponding author).

It is important to highlight that standard LEAS scoring does not incorporate any subjective judgment of accuracy or appropriateness when evaluating the descriptions provided about emotions (although one prior study has explored a measure of appropriateness (Frewen et al. 2008); described below). Further, as stated above, individuals are not told what the goal or aims of the test are. Thus, scores only reflect the granularity and differentiation of the emotion words used to describe self and others, no matter if they match or mismatch with normative responses in the hypothetical scenarios. Therefore, unlike several of the measures of emotional intelligence and emotion understanding described earlier, LEAS scores are best understood as measures of typical, as opposed to maximal, ability (Freudenthaler and Neubauer 2007). That is, they indicate the sophistication of emotion concepts an individual has acquired, as well as the individual’s default (trait) tendency to focus on emotions and apply those concepts. This has the potential benefit of capturing daily-life functioning in relation to emotional awareness, where motivation toward maximal performance may be less frequent. This is consistent with one study showing that LEAS scores increase when individuals are incentivized to try their hardest (Ciarrochi et al. 2005). Thus, while one aspect of emotional awareness is a person’s emotion concept repertoire, another is their default tendency to attend to and focus on emotions.

In some cases, scores may also reflect differences in the patterns of bodily-based affective states generated in response to specific situations/events—as opposed to differences in how they are conceptualized/understood (e.g., cases of low LEAS scores found in individuals who appear to lack arousal responses to affective stimuli; see (Smith et al. 2019a)). Recently, we have proposed a model describing how multiple distinct mechanisms (related to potential differences in appraisals, response generation, conceptualization, and selective attention) can produce a low-EA phenotype (Smith et al. 2018c, 2019b, 2019c; Smith 2020)—highlighting the importance of detailed individualized assessment when designing/selecting interventions to improve EA.

The LEAS was originally designed as a 20-item (scenario) instrument and remains the optimal version for the assessment of individuals, as in clinical settings. Completion of the scale typically takes 1–2 min per item. Because time is a major determinant of experimental design, we explored the option of a shorter version, and determined that a 10-item version had adequate reliability for most purposes (see reliability data above). The 20-item scale has been divided in two to create two 10-item versions, A and B, which are ideal for test–retest purposes. If one wishes to study large samples in which the reliability of findings in any given person is not a high priority, a four-item version has been used successfully (Subic-Wrana et al. 2014). 

Administering the hard copy version of the scale requires the presence of an assistant to provide directions and answer questions. Proficient manual scoring takes approximately 10 h of training. Because of the time and cost needed for hard copy administration and scoring, a digital version of the LEAS has been created (eleastest.net) for automated administration and scoring (Barchard et al. 2010a). This can be completed online at the direction of a researcher or a clinician. Scores from the digital version are highly correlated with those from hand-scoring and provide additional metrics to be calculated across the entire protocol. For example, in addition to generating self, other, and total scores for each item as in classic LEAS scoring, other metrics include the ability to score and sum all emotion words used across the entire protocol as an index of emotional range, identifying implicit (level 1 and 2) and explicit (levels 3, 4 and 5) descriptions within a given response (which may be an indication of the tendency to connect bodily experiences with conceptual processes) and total word count. 

Total word count has ranged from no relationship with LEAS scores (Lane et al. 1990) to positive relationships with moderate effect sizes (*r*s = 0.48 to 0.71; Barchard and Picker 2018), which is to be expected because, by definition, reporting blends of emotions requires using more words. As a separate measure, total word count has received less attention in studies to date; however, when individuals also describe granular emotions in their responses, a greater word count might provide a measure of the degree of overall reflection and effort in considering their emotions (a trait that would be expected to facilitate EA). In other words, as discussed previously (Barchard and Picker 2018), when a person is asked how they feel, being able to provide a lengthy answer is itself an indicator of emotional awareness. However, this measure is not redundant with standard LEAS scores, which still show significant partial correlations with other emotion-related measures after accounting for word count (Barchard and Picker 2018).

There are additional theoretical reasons for caution when considering statistical control of word count or verbal ability in studies using the LEAS, due to the close relation between language and reportable awareness (Weiskrantz 2000). With regard to emotion specifically, recent evidence suggests that verbal reports are not simply a readout of emotional experience but can also play an important role in shaping it (Satpute et al. 2020). Thus, controlling for verbal measures may remove substantive variance from the variable under investigation.

Digital scoring also enables the application of the LEAS scoring algorithms to text samples. For example, in a study of breast cancer patients, participants were asked to spend 20 min writing about their deepest thoughts and feelings about having breast cancer. These essays were scored using the digital scoring engine for LEAS called the Program for Open-Ended Scoring (POES) (Barchard et al. 2010a). The cumulative score of unique scorable emotion words in the essay correlated significantly with that person’s 10-item LEAS score (Lane et al. 2012). To date, POES is only available in English but translations to other languages are planned.

It is recommended that subjects complete the LEAS in their native language because correlations with other variables were higher among native speakers compared to those who completed the LEAS in a language in which they were less fluent (Lane et al. 1995). To date, the LEAS (or children’s version, see below) has been translated into Arabic, Brazilian Portuguese, Chinese, Croatian, Czech, Dutch, French, German, Greek, Hebrew, Italian, Japanese, Korean, Persian, Portuguese and Spanish.

In certain contexts, a participant’s ability to write or type may not adequately capture their ability to conceptualize and describe emotion. An oral version was developed for use in prisoners and found to correlate adequately with the written version (Roberton et al. 2013). Further study is needed to determine if the social context of oral administration systematically affects scores in certain contexts (e.g., social phobia or other psychopathology).

It is also recognized that in certain contexts, there is a need to assess how people structure their emotional experience without explicitly asking them to describe how they would feel in different situations. To address this, we have used the Frith-Happe Animations Task (Castelli et al. 2000), consisting of 30-s animations of moving triangles that depict either random movement, goal-directed activity such as dancing, or more complex “theory-of-mind” animated vignettes that depict complex social interactions such as “coaxing” or “mocking.” Participants view the animations and then verbally “describe what happened.” Although the goal-directed and theory-of-mind animations contain emotional content, neither the stimuli nor the instructions convey that emotion is a focus of attention. Nevertheless, the verbal descriptions of what happened can be transcribed and scored for emotional content using the LEAS scoring system. In a study comparing patients with functional somatic syndromes or conversion disorder to medical patients with somatic symptoms well explained by objective medical findings (control subjects), the animation data revealed that the control subjects spontaneously perceived emotional content even when it is not there (e.g., in random movement), whereas the other two patient groups did not—consistent with impaired prediction of the presence of emotional content in social contexts in the latter participants (Stonnington et al. 2013). 

A children’s version of the LEAS has been developed (LEAS-C; Bajgar et al. 2005) and used in a variety of studies, including some with substantial sample sizes (Agnoli et al. 2019; Mancini et al. 2013; Veirman et al. 2011). The scenarios have been modified from the original adult version to portray scenes that are more age-appropriate (e.g., school-related rather than occupational scenes). This scale shows good psychometric properties. A common finding parallel to that in adults is that in children as young as 10 years of age, girls score higher than boys. 

It is also noteworthy that the LEAS has been adapted to an ecological momentary assessment context to examine variations in state-related emotional awareness (Versluis et al. 2018). Participants were signaled six times per day for two days to speak into a smartphone and describe what they and another person were feeling at the moment (or during the most recent social interaction). The transcribed descriptions were scored and correlated 0.7 with the 10-item LEAS. Importantly, 50% of the variance was state-related, consistent with the theory that emotional awareness has both state and trait characteristics.

### 3.2. Construct Validity

With respect to construct validity, emotional awareness is expected to influence functioning at both the individual and interpersonal/social levels. As exemplified in the opening vignette of this paper, skill at the individual level can make it possible to function more adaptively as a social being, both by being better able to meet one’s own goals in a social context as well as facilitating the maintenance or pursuit of satisfying relationships. As we now review, a variety of studies (but not all; e.g., Waller and Scheidt 2004) support the construct validity of the LEAS—mainly with respect to its status as an individual difference variable. There are, however, both established and recent findings that support its expected relevance in interpersonal/social contexts. However, given the nature of socio-emotional skills, it is at times difficult to fully disentangle whether a given finding is more relevant to an individual or social level of analysis. 

Major examples of results establishing construct validity include studies showing that higher scores on the LEAS are associated with a greater range and differentiation of emotional experiences (Kang and Shaver 2004), greater openness to experience (Ciarrochi et al. 2003; Lane et al. 1990), greater attention to emotion (Lumley et al. 2005), higher scores on perceiving emotions in stories and higher scores on understanding emotions (Ciarrochi et al. 2003), as well as higher scores on describing emotional blends and recognizing emotional progression in stories (Barchard and Hakstian 2004). These and other correlations listed below are typically statistically significant, but of small-to-moderate magnitude, also supporting discriminant validity (i.e., that the LEAS is not measuring the same thing as these other variables). 

The LEAS correlates moderately positively with two cognitive-developmental measures, the Sentence Completion Test of Ego Development (Loevinger and Wessler 1970; Loevinger et al. 1970) and the cognitive complexity of the description of parents (Blatt et al. 1979). Interestingly, these correlations account for separate sources of variance in the LEAS. These findings are consistent with a cognitive-developmental model that highlights the transition from focusing on external/physical to internal/psychological characteristics, the tendency for greater conceptual complexity in the description of emotion, and increasing self–other differentiation. All of these results support the claim that the LEAS is measuring a cognitive-developmental continuum (Smith et al. 2019d). 

Greater emotional awareness is associated with greater cardiac parasympathetic tone, as indicated by heart rate variability at rest (Verkuil et al. 2016), greater differentiation in somatic symptom reporting (Lane et al. 2011), and greater self-reported impulse control (Bréjard et al. 2012; Smith et al. 2021c), all of which are consistent with the theory that functioning at higher levels of emotional awareness (Levels 3–5) modulates function at lower levels (i.e., visceromotor function and somatic experiences at Level 1, and actions and action tendencies at Level 2; (Lane 2000)). Together these findings suggest that LEAS scores capture the construct of levels of emotional awareness—where these levels are envisioned as a nested hierarchy associated with different levels of integration and differentiation.

As further experimental support, one study observed that individuals with lower LEAS scores rated their general sense of well-being as lower during induction of sad mood (compared to baseline ratings), whereas those with higher LEAS scores had stable ratings of general well-being independent of their momentary mood (Ciarrochi et al. 2003). This finding highlights how the LEAS may capture the ability to establish and recall mental representations of one’s own typical emotional state, independent of the immediate circumstances or emotions. In other recent studies, the LEAS was found to correlate positively with measures of general reflective cognition (Smith et al. 2021c), consistent with the domain-general contributions to emotional awareness described above. Relatedly, another study found that the LEAS correlated positively with several indices of cognitive and affective theory of mind, including several subscales of the Mental State Stories task as well as total scores on the “Reading the Mind in the Eyes Test” (Lane et al. 2015a).

Women show consistently higher scores on the LEAS than men, even when controlling for verbal ability (Barrett et al. 2000); a similar pattern is observed in children using the LEAS-C (Mancini et al. 2013). This is consistent with sex differences found for other socio-emotional measures (e.g., Allen et al. 2015; Christov-Moore et al. 2014; Fischer et al. 2018; Wright et al. 2017), and with evolutionary theories suggesting that women on average may have adapted to be more sensitive to internal and external emotion cues than men (Smith et al. 2020b). These results could also reflect differences in early socialization of boys and girls that are present across many cultures (such as encouraging emotional expression in girls but not boys; e.g., see (Chaplin et al. 2005)). When incentives are given to subjects to try their hardest at completing the LEAS, both groups improve, but the sex difference persists (Ciarrochi et al. 2005). 

Greater emotional awareness is also associated with greater emotion recognition ability in faces, scenes, and written descriptions of scenarios (Lane et al. 1990, 1996, 2000b; Wright et al. 2017). In addition, the LEAS is positively associated with empathy ability (Ciarrochi et al. 2003), the tendency to seek help for emotional problems, the actual amount of social support that a person has (Barchard and Hakstian 2004), and greater interpersonal closeness (Lumley et al. 2005). Finally, in a large sample of women with breast cancer (n = 460), higher emotional awareness was associated with greater satisfaction and security in interpersonal relationships (Weihs et al. 2012, March). This potentially contrasts with the findings of a smaller study of 56 heterosexual couples using verbal administration of the LEAS, in which higher emotional awareness was negatively related to the quality of the spousal relationship in women, but no such association was observed in men (Croyle and Waltz 2002). Interestingly, marital dissatisfaction in women was greatest when their LEAS scores were higher than their husband’s and declined as the discrepancy in LEAS scores narrowed—consistent with the potential challenges noted above if higher LEAS scores are discrepant with one’s social context.

### 3.3. Discriminant Validity

With respect to discriminant validity, the LEAS shows small-to-moderate correlations with measures of verbal ability, which are not of sufficient magnitude to obscure the sex difference in LEAS noted previously. Except for a correlation with openness to experience (Lane et al. 1990), correlations with other Big 5 personality variables are typically not observed (Ciarrochi et al. 2003)—although a positive correlation with extraversion has been reported in a Japanese sample (Igarashi et al. 2011). The LEAS consistently fails to show correlations with either trait affect intensity or indices of negative affect, which is an advantage relative to the Twenty-Item Toronto Alexithymia Scale (TAS-20) in the context of assessing medical or psychiatric disorders (Lane et al. 2020b). The correlation with TAS-20 is typically negative and of low magnitude (Maroti et al. 2018), which is likely due to a combination of their differing formats (performance vs. self-report) as well as content differences (Lane et al. 2020b). Indeed, the LEAS tends not to covary significantly with other self-report emotion ability measures (Lumley et al. 2005). Together, these findings suggest that the LEAS is not redundant with other available measures.

### 3.4. Neuroimaging Studies

A series of functional neuroimaging studies have also generated data supporting the validity of the LEAS and the levels of emotional awareness construct. The first functional neuroimaging study involved positron emission tomography (with adequate spatial and temporal resolution, but exposure to very low levels of radiation) and demonstrated that, during the experimental activation of emotional states in the scanner, those subjects who scored higher on the LEAS activated the dorsal anterior cingulate cortex to a greater extent—an area involved in executive control of attention and motor responses (Lane et al. 1998). This finding suggested that emotion may be more likely to engage attention in those who are more emotionally aware. This finding was replicated in a larger study demonstrating that this greater engagement of the dorsal anterior cingulate cortex occurred in the context of higher affective arousal, suggesting that individuals who are more emotionally aware may be better able to tolerate and consciously process more intense emotions than those who are less aware (McRae et al. 2008). 

Another early study involving functional magnetic resonance imaging (fMRI; allowing better spatial and temporal resolution and no exposure to ionizing radiation) observed that in subjects who had experienced traumatic stress but did not have PTSD, recall of the trauma relative to recall of a neutral experience was associated with greater engagement of the rostral anterior cingulate cortex—an area involved in mentalization of cognitive and emotional states (Frewen et al. 2008). The difference between studies in brain areas activated may be attributable to the nature of the tasks performed and the temporal and spatial resolution of the imaging methods used. This is also consistent with another fMRI study that found activation in the rostral anterior cingulate during attention to emotion (Smith et al. 2014).

The psychometric data cited above suggest that people who score higher on the LEAS are more sensitive to emotional cues. This leads to the prediction that greater emotional awareness is manifested in a more robust response to subliminal emotional stimuli. In a behavioral priming study, higher scores on the LEAS were associated with greater priming effects for both verbal and nonverbal (facial) stimuli (Suslow et al. 2001). In a subsequent fMRI study (Lichev et al. 2015), higher emotional awareness was associated with stronger affective reactivity and more activation in brain areas involved in emotion processing when participants were presented with masked happy facial expressions, which the authors suggested might indicate an enhanced positive affective resonance to others at automatic levels of processing.

More recently, we reported on the structural and functional MRI correlates of emotional awareness in 26 healthy participants. As a function of emotional awareness, structural MRI revealed increased cortical thickness in limbic but not default network structures (Smith et al. 2018a), whereas a resting state fMRI study revealed greater connectivity within the default mode and salience networks (Smith et al. 2017a) as well as more efficient global integration across the brain in graph theoretic analyses (Smith et al. 2018f). These findings highlight that changes in the brain, as a function of emotional awareness, are both related to emotion and involve greater connectivity with brain areas involved in emotion processing—and that greater connectivity occurs not only in relation to the default network but throughout the brain as well.

Subjects also completed an emotional working memory task involving the maintenance of subjective emotional content, visual content, bodily sensation content, or no content (“rest”) after exposure to emotion-provoking images (Smith et al. 2018d, 2018e). Results revealed greater activity in the rostral/dorsal anterior cingulate cortex (rdACC) during emotion relative to visual working memory content, a difference that correlated positively with emotional awareness. Even more persuasively, evidence of the presumed neural substrate of the hypothesized psychological processes involved in development of emotional awareness came from another observation in the same study. Namely, the contrast between holding emotions vs. bodily sensations in working memory—highlighting areas of the brain that are more active as a function of emotion above and beyond brain activity due to bodily sensation alone—revealed activity in the rdACC that varied in its magnitude (correlated positively) as a function of emotional awareness. A plausible interpretation of this finding is that greater emotional awareness involves greater conceptual remapping of implicit bodily responses through greater engagement of rdACC and the structures with which it is connected.

We also observed that maintaining the emotional feelings of others in working memory, even when accounting for neural activation attributable to maintaining emotional images/words, activated a left lateral frontal–parietal network (including the anterior insula and posterior dorsomedial frontal cortex), and that this activation correlated positively with emotional awareness (Smith et al. 2017b). Together these and other findings support the hypothesis that greater awareness of one’s own emotional responses is associated with greater engagement of structures involved in interoception, attention, emotional working memory and metacognition (e.g., holding information in mind and reflecting on it), as well as social cognition.

The one neuroimaging study that specifically evaluated emotional awareness during social cognition involved participants viewing valenced (affiliative, aversive, and neutral) animated scenarios of simple ball-like figures while attending either to social or spatial aspects of the interactions (Tavares et al. 2011). Higher LEAS scores correlated with enhanced processing in a left temporal polar region implicated in detailed semantic knowledge. Higher LEAS scores were also associated with a diminished effect of experimentally-manipulated social attention, perhaps because more emotionally aware subjects automatically attend to emotionally relevant stimuli regardless of task instructions. Higher emotional awareness was also associated with increased differentiation of brain activity between animations of differing valence. Decreased emotional awareness, however, was associated with increased activity in regions of pre-motor cortex, consistent with differential neural coding of emotion in semantic vs action systems, which is also consistent with theory.

### 3.5. Studies on Relations to Mental and Physical Health

Lower LEAS scores relative to comparison groups have been observed in mental health conditions, including borderline personality disorder (Levine et al. 1997), somatoform disorders (Subic-Wrana et al. 2005), eating disorders (anorexia and bulimia) (Bydlowski et al. 2005), PTSD (Frewen et al. 2008), depression (Berthoz et al. 2000; Donges et al. 2005), and schizophrenia (Baslet et al. 2009). In the domain of addiction, lower LEAS scores have been observed in those with drug (principally opiates and marijuana) and alcohol dependence (Carton et al. 2008), smokers seeking help with cessation (Carton et al. 2010), and cocaine abuse with impaired insight (Moeller et al. 2014). A challenge for the future will be to determine whether the nature of lower emotional awareness differs between clinical groups, whether low emotional awareness is a common risk factor for psychopathology and addictive disorders in adults, (as has been postulated for adolescents Weissman et al. 2020), or is a consequence of such disorders. 

A recent retrospective study on emotional awareness and early adversity in a student population offers support for the notion that low emotional awareness may be a common risk factor (Smith et al. 2021c). Specifically, LEAS scores were found to be lower in those with greater early childhood adversity (abuse, neglect, low emotional support from caregivers)—where early adversity is known to increase risk for psychopathology (e.g., see Bifulco et al. 2002; Burke et al. 2017; Bush et al. 2016; Taylor et al. 2011). In a clinical sample, Herrmann et al. (2018) observed that, relative to healthy controls, LEAS scores were lower in an inpatient psychiatric sample in whom 62% had early adversity. Those patients in the lowest quartile of LEAS scores had more physical abuse than those in the highest quartile. However, LEAS scores did not differ between patients rated as having clinically significant vs. nonsignificant levels of abuse. In the only study of attachment style to date, lower emotional awareness was associated with disorganized attachment style Subic-Wrana et al. (2007). To date, however, no prospective studies have been undertaken to distinguish low LEAS scores as a risk factor vs. a consequence of psychopathology.

In the physical health domain, lower LEAS scores have been associated with essential hypertension (Consoli et al. 2010), skin-restricted lupus erythematosus (Jalenques et al. 2018), psoriasis (Consoli et al. 2006), and pain on a typical day in irritable bowel syndrome (Smith et al. 2020a)—consistent with greater experience and expression of the somatic aspects of emotion at lower levels of emotional awareness. Relatedly, the LEAS has also been used to demonstrate that functional somatic syndromes and functional neurological disorders are associated with deficits in affective theory of mind (Lane et al. 2015a; Stonnington et al. 2013; Subic-Wrana et al. 2010). 

### 3.6. Clinical Intervention Studies

To date, five studies have demonstrated statistically significant increases in LEAS scores as a result of clinical interventions. One study in 19 patients with traumatic brain injury (TBI) tested interventions focused on training either facial affect recognition or inferring emotions in stories (Radice-Neumann et al. 2009). Both interventions improved LEAS scores for self-focused awareness, while only the facial affect recognition training improved LEAS scores for other-focused awareness. A second study in 13 TBI patients with alexithymia tested an eight-lesson intervention including components associated with psycho-education, emotional vocabulary, labeling/differentiating emotions, interoceptive awareness, and distinguishing emotions from sensations, thoughts, and actions (Neumann et al. 2017). LEAS total scores were significantly higher after the training and remained so at a 3–4-month follow-up visit. A third study in 72 fibromyalgia patients tested a four-session group therapy intervention focused on training adaptive psychological attribution and emotional awareness/expression (Burger et al. 2016). LEAS total, self-focused, and other-focused scores were significantly greater after the intervention, with the strongest effect size for other-focused awareness scores.

While the three studies above were specifically designed to increase emotional awareness, two others also collected LEAS data when testing the efficacy of previously established treatment modalities: art therapy in individuals with schizophrenia (58 inpatients; Montag et al. 2014) and a multi-modal treatment for somatic symptom disorder (394 patients within a psychosomatic ward; Subic-Wrana et al. 2005). While these studies were not designed to improve emotional awareness per se, each study found post-treatment increases in LEAS scores. In the first study, improvements in individuals with schizophrenia (relative to treatment as usual) were found for both self- and other-focused LEAS scores, but only the change in other-focused scores remained significant after accounting for verbal IQ. In the second study, LEAS total scores improved over the course of treatment, independent of measures of negative affect. 

In a recent study, a sixth intervention was completed in 234 healthy volunteers, consisting of a 10-hour online course designed to increase emotional intelligence (Smith et al. 2021b). Relative to a placebo intervention of equal duration in 214 volunteers, emotional intelligence training led to a significant increase in LEAS scores, while the control intervention showed no change. Finally, in a study of 54 patients with panic disorder randomized to cognitive-behavioral therapy or manualized psychodynamic psychotherapy, higher LEAS scores at baseline predicted greater improvement in both modalities (i.e., greater decreases in Panic Disorder Severity Scale scores; Beutel et al. 2013). These findings indicate that emotional awareness of the client may be a powerful variable that influences psychotherapy outcome. 

The studies described above suggest that emotional awareness can be learned in adulthood. This may be important because promoting emotional awareness appears to be a common goal of psychotherapy treatment independent of modality (Burum and Goldfried 2007), comparable in importance to the therapeutic alliance. Some work also suggests that a bidirectional relationship may exist between a strong therapeutic alliance and greater emotional awareness (Lane et al. 2020a). Given these initial demonstrations that emotional awareness can be improved, and that it may also facilitate efficacy of psychotherapy more broadly, it will be important to extend such research to other disorders and to larger samples.

## 4. Future Directions

In this paper, we have reviewed in detail the original development of, and research to date on, the theory of levels of emotional awareness. Here we will now take stock of the current state of this field of research and consider important directions for future research. As detailed above, when the theory of levels of emotional awareness was created (Lane and Schwartz 1987), the notion that a cognitive-developmental model would apply to emotional experience was unprecedented because it appeared to violate basic assumptions about the separateness of emotion and cognition as distinct categories of mental functioning. Since then, recognition of emotion as a type of cognition (Duncan and Barrett 2007) and the inseparability of emotion and cognition (Lane et al. 2000a; LeDoux and Brown 2017; Pessoa et al. 2019) have gained credibility—as has the concept of embodied experience of thought and feeling more generally (Winkielman et al. 2015). Moreover, interconnected evolutionary, cognitive, developmental, and neurocomputational theories of emotional awareness, consisting of domain-specific and domain-general processes, have been formulated (Smith et al. 2018c, 2019b, 2019c, 2020b; Smith 2020). Substantial empirical validation of many of the original theoretical claims about emotional awareness have been made possible by the creation and development of the LEAS in combination with psychometric, psychophysiological, behavioral, and neuroimaging techniques in healthy participants and a wide variety of clinical groups, including those with mental health or systemic medical conditions. The many findings summarized above also support the concept that emotional awareness is a socio-emotional skill.

Despite this promising level of support from existing research, there are several aspects of the theory that have not been tested or have not received sufficient attention in prior research. These include (but are not limited to) the need to (1) further clarify the relationship between emotional awareness and EI, (2) empirically examine the development of emotional awareness over time, and (3) investigate the specific mechanisms that explain observed associations between emotional awareness and adaptive outcomes. Aside from tests of theory, there are also opportunities to expand and refine assessment approaches, and to consider novel interventions for improving awareness. We address each of these topics in turn.

First, while emotional awareness and EI have strong theoretical connections, more research is needed on whether their associated measures are related. As described in more detail above, only a few studies have examined the relationship between LEAS scores and EI measures. One study found significant but small effect size (*r*s = 0.20 to 0.35), relationships between LEAS and performance-based EI scores (Barchard and Picker 2018). As secondary analyses, another neuroimaging study in 26 participants examined the relationship between LEAS and both self-report and performance-based EI measures—finding nonsignificant positive correlations, but with a similar magnitude to the significant relationships observed in the aforementioned study (*r*s = 0.23 and 0.26, respectively; Smith et al. 2017b). However, this study was underpowered to detect small effect sizes (because these correlations were not the main focus of the study). A third study (Smith et al. 2021a) found a significant positive association between LEAS and a performance measure of the management of emotions of others in social situations (i.e., the STEM-B; Allen et al. 2015), which could be seen as an important component of emotional intelligence. While promising, further research is needed to more thoroughly examine the relationship between LEAS and the range of other available measures of socio-emotional skills. The specific hypotheses to test would pertain to the hypothesized role of emotional awareness as a mediator between emotion recognition (in self and others) and adaptive (cognitive, physiological, and behavioral) responses within emotionally challenging contexts—such as effective emotion regulation and social problem solving (Smith et al. 2018b). Testing this would require gathering multiple measures of emotion recognition, emotion regulation, and social problem solving, and testing mediation models in which LEAS scores mediate the relationships between recognition and responding. At the neural level, dynamic causal modeling approaches in functional neuroimaging (Friston et al. 2013) might also be used to test models of the expected causal interactions between neuronal responses associated with emotion recognition, emotional awareness, and emotion regulation (described in Smith et al. 2018b).

A related question regards the degree to which emotional awareness might itself be considered a domain of intelligence. As reviewed above, the LEAS shows significant (but low effect size) correlations with EI measures, and the concept of emotional awareness is related to older proposals regarding intrapersonal and interpersonal domains of intelligence (Gardner 1983). A few studies have also found (weak-to-moderate) correlations between LEAS and both IQ scores (*r* = 0.27 to 0.62; Smith et al. 2017b, 2021a) and related vocabulary measures (*r*s = 0.15 to 0.38; Barchard et al. 2010a; Barchard and Picker 2018; Lane et al. 1990). There is also an extensive body of work examining the relationship between EI and IQ as a means of testing whether EI itself should be conceptualized as a general domain of intelligence (MacCann 2010; MacCann et al. 2014; Olderbak et al. 2019)—with the strongest associations observed between IQ and EI subscales measuring emotion understanding (i.e., those also most strongly related to LEAS scores; (Barchard and Picker 2018)). The inter-relations between emotional awareness, EI, and IQ are suggestive of convergent validity with respect to a domain of intelligence. However, to examine this idea further will require studies with large sample sizes capable of supporting the factor-analytic approaches that have been applied to address this question with respect to EI. 

Next, there is a need to better understand the mechanisms whereby higher emotional awareness might lead to the improved levels of functioning observed in the studies reviewed above—or to understand more generally why associations exist between emotional awareness and positive mental and physical health outcomes. This overlaps somewhat with the questions discussed in relation to EI above. Namely, while there is considerable theoretical work proposing that higher emotional awareness should facilitate automatic/voluntary emotion regulation strategies as well as improved interpersonal problem solving (e.g., Lane et al. 2015b, 2018; Smith et al. 2018b, 2019b), more empirical work is necessary to test these proposals. If confirmed, this would more directly explain beneficial outcomes. One example of existing work toward this end is a study finding that LEAS scores are positively associated with a performance-based measure of the ability to effectively manage others’ emotions (Smith et al. 2021a)—consistent with the idea that emotional awareness promotes well-being through effective interpersonal problem solving. Another study found that LEAS scores correlated positively with cognitive reappraisal and negatively with emotion suppression (Subic-Wrana et al. 2014). Outside of research on the LEAS specifically, empirical work has also shown that explicitly labeling emotions—which arguably requires awareness—can have adaptive effects on emotion regulation and planning (Vine et al. 2019). Some studies have also linked measures of higher emotion differentiation to greater emotion regulation ability (Barrett et al. 2001) and to reduced tendencies to engage in maladaptive regulation strategies involving aggression, substance use, or self-injury (Kashdan et al. 2015). It is also known that, relative to emotion acceptance (and other adaptive regulation strategies), suppressing emotions increases physiological arousal Gross (1998b), Hofmann et al. (2009), which may negatively impact health if chronic. However, while high emotion differentiation and low emotion suppression are theoretically linked to emotional awareness, the aforementioned relationships are yet to be thoroughly investigated using the LEAS (but see Subic-Wrana et al. 2014). Thus, it will be important to more thoroughly investigate the behavioral and psychophysiological differences, and potential mediation relationships, that explain the association between higher LEAS scores and greater health and well-being.

The third important research direction mentioned above pertains to how the capacity for emotional awareness develops in children. As reviewed above, the theory of levels of emotional awareness predicts that children should show gradual increases in LEAS scores during childhood and adolescence. The level attained is in turn expected to be a function of the quality of socio-emotional interactions with early caregivers and peers. When measured at a given point in time, emotional awareness is also expected to be modulated by state variables—such that LEAS scores are predicted to decrease under states of very low or very high levels of negative affective arousal. If such negatively valenced, high arousal states are chronic in childhood (e.g., as in cases of early adversity), this would be predicted to hinder development of emotional awareness generally. As reviewed above, retrospective work in adults has shown that early childhood adversity is negatively associated with LEAS scores; (Smith et al. 2021c), and cross-sectional work using the LEAS-C in children and adolescence has found the expected positive relationship with age (Agnoli et al. 2019; Mancini et al. 2013; Veirman et al. 2011). However, longitudinal studies have not yet directly examined how emotional awareness develops in childhood using the LEAS-C, or its interaction with state variables (future studies are also needed to test the predicted relationship between LEAS scores and affective arousal generally). There are also many specific details that remain to be addressed about the timing and nature of the particular early social interactions that are most important.

Therefore, building upon decades of research on emotional development more broadly (e.g., Schore 2015; Sroufe 1997), longitudinal research is needed to determine what interactions with caretakers and peers are necessary, and at what age, for adequate if not good emotional awareness to develop. For example, are there sensitive periods where attunement and responsiveness are especially important and other time periods when they are not? If so, how much attunement and responsiveness is needed? How much does preverbal attunement pave the way for the ability to put emotions into words? How much do habits of emotion communication in one’s family environment and peer group limit or permit what is possible in this area? A related issue is whether the same processes and timing apply equally to boys as well as girls (Bath 2020). Importantly, if abuse or neglect occurs, how and when must intervention be made to mitigate its effects? Initial exploratory studies are needed to begin to answer these questions.

Further questions pertain to later developmental stages. For example, longitudinal studies will also be needed to determine how socialization processes in later childhood and adolescence (e.g., in a school setting) interact with the level of emotional awareness first achieved in earlier childhood. It is also important to consider how realistic it is to aspire to enable every child graduating from high school to be capable of the highest levels of emotional awareness—and to consider what cognitive prerequisites may be necessary for attaining such a goal. 

As reviewed above, LEAS scores are positively correlated with several measures of cognitive ability (with weak-to-moderate effect sizes). However, there are also dissociations. In this regard, Baddeley et al. (1997) demonstrated that, despite equivalent performance on traditional tests of frontal lobe function (such as verbal fluency), patients with frontal lobe lesions and socially disrupted behavior were more impaired in their performance in a dual task paradigm (an orally administered digit span memory task performed simultaneously with a visual tracking task) than were patients with frontal lobe lesions but no behavioral impairment. The dual task paradigm taxes working memory and executive functions. Baddeley and colleagues speculated that successful social adaptation requires the “dual task” ability to stay in touch with the needs of others while simultaneously paying due attention to one’s own needs. These findings suggest that the highest levels of emotional awareness may require proficient dual task capabilities more generally—which could be tested in future research. To facilitate application of this ability to real-life circumstances, instruction in levels of emotional awareness functioning might be coordinated with instruction in a cognitive-developmental framework for interpersonal negotiation strategies (Selman 1981), ranging from use of physical force to collaboration in pursuit of mutual goals based on the ability to understand and reconcile the needs, goals, and wishes of both self and other.

Additional areas of future research involve assessment methods themselves. One likely reason for the success of the LEAS over the past 30 years is that the scoring system focused on the structure of reported experience and not its content or appropriateness, thus requiring little or no subjective judgment or inference from raters and promoting reliability in scoring. However, with the advent of natural language processing and other automated methods of meaning assessment (Chowdhary 2020), it is possible to assess additional aspects of participants’ responses—such as the appropriateness or normativity of emotional responses described in each hypothetical scenario. This corresponds not to the differentiation of emotions but to whether they are contextually appropriate. An example is that in contexts such as PTSD, feelings such as shame, embarrassment, and guilt may be experienced and reported in typically pleasurable scenarios, yielding high LEAS scores that are nevertheless maladaptive and indicative of psychopathology (Frewen et al. 2008). Contextual assessment can also aid spelling correction to facilitate use of the digitized glossary that currently requires exact matches in the text for accurate scoring. There is also a need to develop a larger pool of scenarios for test–retest purposes on the internet as well as the creation of self-learning algorithms to expand glossary content. Importantly, current methods have been validated for children as young as eight years of age, but verbal and behavioral measures of emotional awareness are needed for even younger children. Given the need to transcend limitations in reading and writing ability, it would be useful to concretely present age-appropriate scenarios in video format, design software to assess progressive differentiation in facial expressions and gestures and develop software to automatically transcribe and score vocal recordings of verbal responses.

While at present the LEAS affords a total score as well as subscale scores for self-focused and other-focused emotional awareness, recent work has also highlighted the potential opportunity to develop additional subscale scores reflecting three dimensions of emotional awareness inherent in the LEAS (Smith et al. 2018c, 2019d). One dimension corresponds to the use of externally (level 1 and 2) vs. internally (level 3) focused-terms, independent of their granularity. A second dimension corresponds to the granularity of the terms used to describe feelings, whether internally or externally focused. A third dimension is self–other differentiation, which could be assessed on each item independent of both granularity and internal vs. external focus. Specific means of calculating subscale scores for these dimensions based on LEAS item responses have yet to be developed but could offer additional insights about the more specific aspects of emotional awareness that are most developed or most in need of intervention in clinical settings. 

Finally, although it has been demonstrated that various interventions can increase emotional awareness as noted above, a method for doing so that is designed specifically to promote emotional awareness in children as well as adults in nonclinical settings has yet to be developed. A guiding principle is that emotional awareness is like wine tasting (Lane and Pollerman 2002) in that it takes practice to find the appropriate words to assign to sensory experiences. This requires learning the concepts and appropriate vocabulary and having an expert available to calibrate the learning process through repeated feedback. As noted above, the fundamental skill of recognizing and describing the experiences of self and others in differentiated ways must be supplemented with learning how to use this information in different social settings. To avoid boredom and promote motivation, a video game format might be optimal, in which choices for responding to different social contexts are presented, and then feedback is given on the pros and cons of the options presented. The ideal game would promote learning while being fun to play (Hromek and Roffey 2009). A recently developed web-based emotional intelligence training has taken initial steps in this direction (Persich et al. 2021).

## 5. Conclusions

The theoretical background, description of measurement methods, empirical findings, and future research agenda in the area of emotional awareness have been reviewed. The LEAS is a reliable and valid measure that can be applied in individual and social settings. Progress to date can be used as a foundation for future research on developmental processes and milestones in the acquisition of this ability, assessment, and interventions to promote emotional awareness in children as well as adults. Advances in these areas are likely to promote mental as well as physical health, as well as the ability to navigate the complex interpersonal situations needed to succeed socially and occupationally. Goals for such intervention methods would be to promote a greater capacity to understand the emotional experiences of self and others (e.g., affective theory of mind), greater tolerance for differences in lifestyle and cultural background, and a greater ability to cooperate and collaborate, which is arguably the hallmark of civilization and human existence (Tomasello et al. 2005).

## Figures and Tables

**Table 1:** Levels of Emotional Awareness.

Level 1—Somatic sensations/visceromotor activity: Emotional experience at this level consists of bodily sensations. Individuals describe somatic sensations or are unable to provide a description of their experience.
Level 2—Action tendencies/somatomotor activity: Emotional experience at this level consists of actions or action tendencies (approach or avoidance, self-injurious behavior, etc.) and is described similarly. These action tendencies have an associated valence (feeling globally good or bad) that is undifferentiated.
Level 3—Individual feelings: At this level individuals experience emotion as a discrete and specific emotional feeling state. The description of emotion is one-dimensional and often stereotyped (“I feel angry”).
Level 4—Blends of feeling: This level is characterized by the capacity to have feelings that are opposed to or clearly different from each other, e.g., feeling sad yet hopeful.
Level 5—Blends of blends of feeling: At this level the individual has the capacity to appreciate complexity in the experiences of self and other simultaneously. The individual at this level is also able to appreciate the multi-dimensionality and nuance of the other’s feelings by imagining oneself in the other’s situation, unbiased by one’s own emotional state. Comparing the combination of feelings a given person might feel in one situation versus another is another example of level 5 functioning.

**Table 2:** LEAS Scoring.

Terms Used	Level Score
Cognitions: justified, disbelief, attentive	Level 0
Bodily sensations: sick, sleepy, dizzy	Level 1
Action tendency: feel like punching a wall, feel like crying Negative or positive valence (undifferentiated): good, bad, low	Level 2
Specific, discrete emotion: happy, sad, afraid	Level 3
Uses two or more Level 3 words for self or other	Level 4
Both self and other are at Level 4 and terms used are not identical	Level 5

**Table 3:** Internal consistency reliability of the LEAS, computerized LEAS and children’s version of the LEAS (LEAS-C; (Barchard et al. 2011)).

Citation	Participants	Number of Items	Internal Consistency
**LEAS**			
Bydlowski et al. (2002)	121 healthy French adults	20	0.75
Lane et al. (1990)	40 undergraduates	20	0.81
Lane et al. (1995)	51 medical students	20	0.82
Lane et al. (2000b)	379 healthy community members	20	0.88
Ciarrochi et al. (2005)	316 healthy adults	16	0.80
Richard Lane (unpublished data)	375 healthy adult volunteers	10: Part A 10: Part B	0.78 0.79
Barchard and Hakstian (2004) Subic-Wrana et al. (2014)	176 undergraduates 380 healthy adult volunteers	5 4	0.58 0.61
**Computer-Administered LEAS**			
Barchard et al. (2010a)	66 undergraduates	20	0.88
Barchard et al. (2010b)	268 undergraduates	20	0.84
**LEAS-C**			
Bajgar et al. (2005)	51 children (aged 10–11)	12	0.66
Agnoli et al. (2019)	488 children (aged 8–12)	12	0.68
Veirman et al. (2016)	574 children (ages 8–16)	6	0.74
Veirman et al. (2011)	381 children (aged 10–17)	12	0.76

**Table 4:** Inter-rater reliability of the LEAS and LEAS-C (Barchard et al. 2011).

Citation	Participants	Number of Items	Inter-Rater Reliability
**LEAS**			
Lane et al. (1990)	40 undergraduates	20	0.81
Wrana et al. (1998)	331 German medical students	20	0.81
Noland et al. (2005)	66 undergraduates	20	0.97
Lane et al. (1995)	51 medical students	20	0.97
Novick-Kline et al. (2005)	293 undergraduates	20	0.95
Barchard (2009)	48 undergraduates	20	18 raters: pairs ranged 0.72–0.99 (mean 0.94)
Ciarrochi et al. (2005)	316 healthy adults, regular instructions	8	0.92
Ciarrochi et al. (2005)	316 healthy adults, half in control condition and half in high motivation condition	8	0.96
Barchard and Hakstian (2004)	176 undergraduates	5	0.96
Lane et al. (2000b)	379 healthy community members	5	0.96
**LEAS-C**			
Bajgar et al. (2005)	51 children (aged 10–11)	12	0.89
Agnoli et al. (2019)	488 children (aged 8–12)	12	0.71–0.87
Veirman et al. (2016)	574 children (aged 8–16)	6	0.92–0.95

## Data Availability

Not applicable.
